# Bladder prolapse (cystocele) among African, Asian, and Middle Eastern women: a clinical and socio-cultural perspective

**DOI:** 10.3389/fgwh.2025.1696375

**Published:** 2025-11-24

**Authors:** Gayathri Delanerolle, Vindya Pathiraja, Tharanga Mudalige, Nirmala Rathnayake, Abirame Sivakumar, Wijamunige Nimesha Prashadini, Nihal Mohamed Al Riyami, Lamiya Al-kharusi, Mohammad Haddadi, Fred Tweneboah-Koduah, Om Kurmi, George Uchenna Eleje, Peter Phiri, Lauri Romanzi, Sohier Elneil

**Affiliations:** 1 Institute of Applied Research, University of Birmingham, Birmingham, United Kingdom; 2 Clinical Trials Facility, Tom Rudd Unit, Hampshire and Isle of Wight Healthcare NHS Foundation Trust, Southampton, United Kingdom; 3 Faculty of Allied Health Sciences, University of Ruhuna, Matara, Sri Lanka; 4 Faculty of Medicine, University of Jaffna, Jaffna, Sri Lanka; 5 Department of Nursing, General Sir John Kotelawala Defence University, Colombo, Sri Lanka; 6 Obstetrics & Gynecology, Sultan Qaboos University, Muscat, Oman; 7 Tehran University of Medical Sciences, Tehran, Iran; 8 Obstetrics & Gynecology, Narh-Bita Hospital, Tema, Ghana; 9 Faculty of Health and Life Science, Coventry University, Coventry, United Kingdom; 10 Department of Obstetrics and Gynaecology, Nnamdi Azikiwe University, Kigali, Nigeria; 11 School of Medicine, University of Southampton, Southampton, United Kingdom; 12 Obstetrics & Gynecology, Jefferson College of Population Health, Philadelphia, PA, United States; 13 Institute of Womens Health, University College London, London, United Kingdom

**Keywords:** bladder prolapse, cystocele, pelvic organ prolapse, women's health, health systems

## Abstract

Bladder prolapse, also known as cystocoele, is the most common form of pelvic organ prolapse (POP), significantly affecting women's physical, sexual, and psychosocial health, particularly in low- and middle-income countries (LMICs). Despite its widespread prevalence and disabling consequences, bladder prolapse remains under-recognised and inadequately addressed in global health strategies. Prevalence estimates vary widely across regions, from 3%–64.6%, influenced by diagnostic methods and cultural reporting biases. Common risk factors include high parity, early childbirth, prolonged labour, poor postpartum care, malnutrition, obesity, and ageing. Clinical diagnosis often relies on simplified grading systems in resource-limited settings. Conservative treatments like pelvic floor muscle training and pessary use are underutilised due to lack of staff training, and cultural barriers. Surgical management, primarily native tissue anterior repair, is often inaccessible or inconsistently performed. Key challenges include sociocultural stigma, lack of epidemiological data, inadequate provider training, and limited access to specialised care. Bladder prolapse remains a hidden burden in LMICs due to structural, sociocultural, and health system gaps. Addressing it requires integrating prolapse screening into routine maternal care, expanding conservative management, training healthcare providers, reducing stigma, and investing in locally relevant research and national guidelines. Here, we argue for evidence-based practices in LMICs to improve our understanding through epidemiology, risk factors, clinical presentation, diagnostic practices, treatment approaches, and sociocultural barriers. Elevating bladder prolapse as a public health and gender equity issue can improve health outcomes and quality of life for millions of women in Africa, Asia, and the Middle East.

## Background

Pelvic organ prolapse (POP) represents a spectrum of disorders, including cystocoele, rectocele, uterine prolapse, and vaginal vault prolapse, with cystocoele being the most frequently diagnosed form in many regions globally (1, 2). A cystocoele is characterised by the downward displacement of the bladder into the vaginal canal due to weakness or injury to the pelvic floor muscles and connective tissues. It is also known as a bladder prolapse in lay terms, and anterior vaginal wall prolapse in anatomically descriptive academia as it reflects the precise compartment affected and aligns with International Continence Society and International Urogynaecology Association terminology for prolapse (3). For this article, the term bladder prolapse will be used interchangeably with cystocoele.

Approximately 30%–40% of women worldwide experience some degree of pelvic organ prolapse during their lifetimes, with varying degrees of severity and symptomatology (2, 4). Although many cases are mild and asymptomatic, significant numbers of women develop moderate-to-severe symptoms that markedly impair quality of life. These symptoms typically include urinary difficulties such as stress urinary incontinence, urinary urgency, incomplete bladder emptying, pelvic pressure or discomfort, and challenges related to sexual function and psychosocial well-being (5, 6). Such symptoms often lead to diminished self-esteem, social isolation, anxiety, and depression, creating profound emotional and social impacts beyond the physical discomfort (7).

Despite its significant prevalence and the debilitating nature of its symptoms, bladder prolapse remains a neglected and underreported women's health issue in global health discourse. Historically, conditions such as obstetric fistula, maternal mortality, and severe acute maternal morbidity have captured greater international attention due to their dramatic presentations and clear implications for maternal survival (8). By comparison, cystocoele, and other forms of POP, although widespread and disabling, have often been viewed incorrectly as natural and inevitable consequences of childbirth and ageing rather than preventable and treatable medical conditions. Consequently, these conditions have not received comparable attention in global women's health policies, programming, or funding streams (9).

In low- and middle-income countries (LMICs), the situation is particularly acute, as women. Women often experience significant barriers to diagnosis, treatment, and effective management. The stigma surrounding reproductive health conditions, limited healthcare infrastructure, poverty, and sociocultural norms around women's roles and responsibilities compound the problem, resulting in many women enduring years or even decades of untreated symptoms (10). Also, access to qualified healthcare providers, especially urogynaecology or pelvic health specialists, remains limited or non-existent in many rural and underserved regions. This healthcare access disparity exacerbates the burden of prolapse, as women often delay seeking medical assistance until symptoms become severe, thus increasing the complexity and risk associated with treatment (11).

The populations in Africa, South Asia, and the Middle East face distinct but overlapping risk factors and sociocultural contexts contributing to high prolapse prevalence. Common regional factors include high parity (multiple vaginal births), early-age childbirth, inadequate obstetric care during complicated deliveries, prolonged labour, postpartum practices involving heavy physical labour, poor nutritional status, and limited postpartum recovery periods (12, 13). Additionally, changing demographic patterns, such as rising obesity rates in urban areas of the Middle East and South Asia and ageing populations, further exacerbate the risk profile for cystocele and related conditions (13, 14). These demographic transitions mean that the burden of pelvic organ prolapse will likely increase over time unless targeted preventative interventions and improved management strategies are implemented.

Given these complexities, addressing bladder prolapse effectively requires clinical interventions and a deeper understanding of the epidemiological landscape, sociocultural contexts, and healthcare systems in these regions. This commentary provides a critical review and synthesis of existing literature regarding bladder prolapse in selected LMIC populations from Africa (Nigeria, Kenya, Rwanda, Ghana), South Asia (India, Nepal, Sri Lanka), and the Middle East (Oman, Jordan, Lebanon, UAE). Specifically, this review aims to clarify regional epidemiological patterns, explore current clinical practices and gaps, analyse sociocultural barriers impacting care-seeking behaviours, and propose targeted strategies for improving prevention, diagnosis, and treatment. By highlighting challenges and actionable opportunities, this commentary aims to inform future research agendas, clinical practice improvements, and policy developments and, ultimately, to contribute toward better health outcomes and quality of life for women affected by bladder prolapse across these diverse regions.

### Epidemiology and risk factors

POP, particularly bladder prolapse or cystocoele, exhibits significant epidemiological variation globally, influenced by diverse demographic, socioeconomic, and cultural factors. Epidemiological estimates indicate marked regional disparities in prevalence, largely attributable to methodological differences in study design, populations surveyed, diagnostic criteria, and sociocultural reporting biases. Clinical examination-based studies consistently report higher prolapse prevalence rates (20%–60%) compared to symptom-based self-reporting methods (3%–15%), revealing a considerable “hidden burden” of undiagnosed prolapse (6, 7). Such discrepancies underscore the importance of active clinical screening to fully appreciate prolapse's true epidemiological impact.

### South Asia

In South Asia, POP is a prevalent public health issue, particularly prominent in rural and socioeconomically disadvantaged communities. In Nepal, national estimates indicate symptomatic prolapse prevalence around 10%; however, localised studies in rural and remote districts report significantly higher rates, with prevalence as high as 60.9%, predominantly involving cystocoele (8, 9). This elevated burden is strongly associated with sociocultural practices such as early marriage, frequent and closely spaced pregnancies, home childbirths without skilled assistance, and immediate resumption of heavy agricultural labour postpartum (9, 10).

Similarly, India exhibits significant regional variations in reported prolapse prevalence, ranging from approximately 3%–20%, depending on diagnostic methods, geographical locations, and urban-rural divides (10). Hospital-based studies frequently identify higher prolapse prevalence among women attending gynaecological services compared to community self-report surveys, reflecting limited community awareness, stigma, and barriers to accessing healthcare (10, 12). In Sri Lanka, comprehensive community-level epidemiological data remain scarce; available clinical reports suggest substantial underreporting and hidden morbidity comparable to other South Asian contexts, influenced by similar sociocultural and healthcare access barriers (13).

### Sub-Saharan Africa

Sub-Saharan African studies also reveal extensive regional and methodological variations in prolapse prevalence. In Ethiopia, community-based studies identified prevalence estimates ranging from approximately 23.5% to over 56% in certain rural populations (12). These high prevalence figures correlate closely with cultural practices around childbirth, including limited access to skilled obstetric care, high parity, prolonged labour, and arduous postpartum physical activities (12).

In Tanzania, a landmark population-based survey in rural Kilimanjaro reported an alarming prolapse prevalence of 64.6%, predominantly involving the anterior vaginal wall (cystocoele) (14). Such findings starkly contrast with Nigerian community-based surveys reporting significantly lower prevalence (around 6.5%) (15). However, lower Nigerian figures are likely reflective of substantial underreporting due to cultural stigmatisation, lack of awareness, and limited health-seeking behaviours rather than a genuinely lower incidence. Clinic-based Nigerian studies, representing women seeking healthcare, further underscore the potential underestimation of the true prolapse burden (15). Thus, prolapse remains markedly underreported and inadequately recognised across sub-Saharan Africa.

### Middle East

In the Middle East, POP prevalence remains considerable, influenced historically by high fertility rates, recent shifts toward urbanisation, increasing obesity, and changing lifestyle patterns. Early epidemiological data from Jordan identified prolapse prevalence of approximately 34.1%, predominantly involving cystocoele, highlighting significant health system implications for women's reproductive health management (16). Amongst 482 Emirati women, 29.6% of the cohort reported prolapse symptoms though the type of prolapse was not determined (16). Of note 73% of these women were affected by vaginal soreness associated with the menopause, and one third had to digitate to empty their bladder or bowel indicating significant health implications for the women.

In rural Egypt, prolapse prevalence has been documented at approximately 56.3%, predominantly anterior compartment prolapse (17). These high prevalence rates correlate with early marriage, multiple childbirths, limited healthcare access during childbirth, and insufficient postpartum recovery periods. Recent research from the United Arab Emirates (UAE) documented symptomatic prolapse prevalence approaching 30% among middle-aged Emirati women, exacerbated notably by rising obesity rates, historically high fertility, and increasing longevity (17). Gulf countries, including Oman, Lebanon, and the UAE, face demographic transitions characterised by declining fertility yet persistently high prolapse prevalence among older cohorts who experienced higher parity. Additional risk factors such as gestational diabetes and the resultant macrosomia infants further exacerbate prolapse risk in this region (18).

### Common risk factors across regions

Several common risk factors consistently emerge across African, Asian, and Middle Eastern populations, contributing significantly to the prolapse burden, as outlined in Supplementary Table S1 in the supplementary.

Understanding these complex epidemiological patterns and interconnected risk factors provides a critical foundation for developing targeted prevention and management strategies. Addressing these common yet modifiable risks through culturally sensitive interventions, public health education, improved maternal healthcare access, and early prolapse detection initiatives could substantially mitigate the regional and global prolapse burden.

### Clinical presentation and diagnosis

Women with bladder prolapse typically present with a spectrum of clinical symptoms, which significantly vary based on individual prolapse severity, duration, and associated conditions. Commonly reported symptoms include a sensation of vaginal bulging or heaviness (“something coming down”), pelvic discomfort or pressure, urinary symptoms such as stress urinary incontinence (leakage with coughing, sneezing, or physical exertion), urinary urgency, frequency, difficulty initiating or maintaining urination, incomplete bladder emptying, and recurrent urinary tract infections (UTIs). Additionally, many affected women experience discomfort or pain during sexual intercourse (dyspareunia), significantly affecting sexual health and intimate relationships (1, 19).

Importantly, symptom severity does not consistently correlate with the anatomical severity or stage of prolapse. Some women report significant discomfort and functional impairment even with relatively mild anatomical prolapse. In contrast, others tolerate advanced-stage prolapse with minimal complaints due to cultural stigma, misinformation about available treatments, or normalization of symptoms as an expected consequence of childbirth and ageing (5, 20). Indeed, qualitative studies from LMICs indicate that many women suffer silently for years before seeking medical attention, often due to embarrassment, stigma, or resignation that prolapse is inevitable (10, 20).

In resource-limited regions, prolonged neglect frequently results in women presenting at advanced stages, often accompanied by complications such as vaginal ulceration, chronic UTIs, and severe stress or overflow incontinence. Clinically significant prolapse may protrude visibly beyond the vaginal introitus, leading to considerable physical and psychological distress, social isolation, and reduced mobility (5, 10).

### Diagnostic approaches

Clinical evaluation remains the cornerstone of cystocele diagnosis. The gold standard for assessing prolapse severity and compartment involvement is the POP Quantification (POP-Q) system. POP-Q systematically measures the descent of various vaginal anatomical landmarks relative to the hymenal ring during a standardised pelvic examination. This method categorises prolapse into five stages (Stage 0: no prolapse, through Stage IV: complete vaginal eversion), providing reliable, reproducible staging useful for clinical management and research comparisons.

However, due to its complexity and the requirement for specific training, the POP-Q system is less commonly employed in rural and resource-limited settings. Instead, simpler descriptive grading systems, categorising prolapse into first-degree (mild descent within the vagina), second-degree (descent at or near vaginal opening), and third-degree (complete prolapse outside the vaginal introitus), remain widely utilised due to practicality and ease of use. Despite being less precise, these simpler systems facilitate diagnosis, triage, and basic management in areas where specialist training is limited.

### Assessment for associated conditions

Comprehensive cystocoele diagnosis requires evaluating the patient for commonly associated complications, particularly urinary tract infections and stress urinary incontinence (21). Urinalysis or culture should be performed routinely when women report urinary symptoms, given the high prevalence of urinary retention and resultant UTIs in moderate-to-severe prolapse. SUI assessment typically involves clinical “stress testing,” where leakage is observed during coughing or straining manoeuvres with a comfortably full bladder. In some cases, advanced prolapse can mask underlying SUI (occult stress incontinence), becoming evident only upon prolapse reduction, often requiring careful preoperative evaluation.

### Additional diagnostic considerations

In specialised urban or tertiary care settings within LMICs, additional diagnostic modalities such as dynamic pelvic floor ultrasound, magnetic resonance imaging (MRI), or urodynamic studies may occasionally be used for complex prolapse cases or unclear clinical presentations. These imaging methods offer detailed anatomical visualisation of prolapse extent, compartmental involvement, and concomitant pelvic floor disorders (e.g., enterocele, rectocele, urethral hypermobility). However, their application remains limited by excessive costs, technological availability, and operator expertise, making them largely inaccessible in most rural or under-resourced settings.

Given the pervasive underreporting and normalisation of prolapse symptoms, proactive clinical screening for cystocoele, particularly during routine gynaecologic exams, postpartum assessments, and menopausal health checks, is strongly recommended. Training primary care providers, midwives, and nurses to conduct basic prolapse screening and symptom inquiry can substantially improve early identification, facilitate timely management, and reduce the prevalence of advanced complications.

### Treatment and management strategies

Effective management of cystocoele should be individualised, balancing prolapse severity, and patient-specific factors such as age, comorbidities, reproductive plans, and available healthcare resources. Management strategies broadly divide into conservative (non-surgical) and surgical approaches, ideally following a stepwise model emphasising conservative measures first, progressing to surgery when symptoms persist or worsen, as outlined in Supplementary Table S2 in the supplementary.

### Sociocultural barriers to care

Women frequently experience profound stigma and shame associated with cystocoele, driven by misinformation, gender norms, and patriarchal beliefs linking prolapse to moral inadequacies or sexual dysfunction (20, 22). Cultural modesty necessitating female healthcare providers, lack of decision-making autonomy, financial dependence, and prioritisation of family duties further delay care-seeking (23). Traditional normalisation of prolapse as inevitable after childbirth contributes significantly to delayed diagnosis and treatment (5). This is further complicated in women affected by the menopause.

Women experiencing menopausal symptoms, particularly those related to urinary and sexual health, face substantial emotional, cultural, and social barriers that impede timely access to medical care. Feelings of embarrassment, shame, and vulnerability are common when addressing intimate health concerns, often leading to reluctance in discussing these issues with healthcare providers, family members, or peers (24, 25). This internalized hesitation is compounded by broader societal stigma, where conditions such as urinary incontinence, pelvic organ prolapse, and sexual dysfunction are frequently perceived as taboo or natural, inevitable aspects of aging that women are expected to endure rather than seek treatment for (26–28). Research has demonstrated that stigmatization not only discourages open dialogue but also perpetuates misinformation and normalizes suffering, exacerbating health inequities among midlife women (29).

Cultural expectations of modesty, which necessitate the availability of female healthcare providers, combined with a lack of decision-making autonomy, financial dependence, and the prioritization of family duties, often result in substantial delays in care-seeking (27, 30). In many communities, prolapse is traditionally normalized as an inevitable consequence of childbirth, contributing significantly to the acceptance of symptoms and delays in both diagnosis and treatment (27, 31).

Consequently, by the time women seek medical attention, their symptoms are often significantly advanced, complicating management pathways and negatively impacting their physical functioning, sexual health, psychological well-being, and overall quality of life (32, 33). Addressing these challenges requires a multifaceted approach involving public education to normalize discussions of menopausal and pelvic health, proactive and sensitive engagement by healthcare providers, and broader stigma reduction strategies to promote early diagnosis and timely intervention.

### Gaps in research and clinical practice

Despite the recognised burden and significant morbidity associated with bladder prolapse (cystocele), considerable gaps in both research and clinical practice persist across many low- and middle-income countries (LMICs). These gaps substantially limit effective care delivery, accurate disease prevalence and severity assessment, and informed policy formulation. A major contributor is the inadequate training of primary healthcare providers—including nurses, midwives, and general practitioners—in recognising and managing pelvic organ prolapse. Although frontline healthcare workers are ideally placed to detect early-stage prolapse and initiate conservative measures such as pelvic floor muscle training (PFMT) or pessary fitting, their training in these critical skills is often superficial or absent. As a result, opportunities for early intervention are frequently missed, leading to disease progression and increased healthcare burden.

These clinical deficits are compounded by pronounced disparities in access to services, particularly between rural and urban areas, and (34) between the government and private healthcare sectors. In rural and resource-limited settings, women may have little or no access to specialist care, while private providers, where available, often offer better services but at unaffordable costs. Underpinning these systemic issues is a chronic funding gap in women's health services in LMICs, where prolapse and other urogynaecological conditions receive limited attention in national health priorities. To address these multifaceted barriers, urgent investment is needed in capacity building, health systems strengthening, and equitable service provision across all levels of care.

#### Limited robust epidemiological data and standardised diagnostic methods

One of the most critical gaps in addressing cystocele in Africa, Asia, and the Middle East is the scarcity of reliable, comprehensive epidemiological data. Current prevalence estimates vary widely due to differences in study methodology (clinical examination vs. self-report), diagnostic criteria, and study populations. Without robust epidemiological data, it remains challenging to accurately quantify the true public health burden of cystocele, hindering resource allocation, priority setting, and targeted policy interventions. Additionally, variability in diagnostic methods—from the detailed POP Quantification (POP-Q) system to simplified first-to-third-degree grading—creates inconsistencies in data comparability across different regions (35).

#### Inadequate frontline healthcare worker training and inequitable access to services

A significant clinical practice gap in LMICs involves inadequate training among primary healthcare providers—including nurses, midwives, and general practitioners—in recognising and managing POP. Although frontline healthcare workers are ideally positioned to identify early-stage cystocele and initiate conservative management such as pelvic floor muscle training (PFMT) or pessary fitting, their training in these areas is often minimal or entirely lacking (34). As a result, many women miss the opportunity for early diagnosis and timely intervention, often progressing to more advanced stages that require complex and costly surgical management. Addressing these challenges requires investment in targeted training programmes for frontline providers and systemic improvements to ensure equitable access to women's health services across all regions and care sectors, a shortage of specialised urogynaecology services, and inconsistency in surgical care quality.

Specialised urogynaecology care remains scarce in most resource-limited settings, creating a critical gap in prolapse management. Few clinicians possess formal training in urogynaecological surgery or advanced conservative management, significantly impacting the quality of available surgical interventions and patient outcomes. Furthermore, even in places where surgical services are available, variability in surgical skills, techniques, and perioperative care results in inconsistent outcomes and higher complication rates, undermining trust and contributing to patient reluctance to seek care. Expanding specialised training programs and standardising surgical practices through accredited training programs are key areas for future clinical development (36).

#### Lack of comparative effectiveness research tailored to LMIC contexts

A major research gap is the limited availability of comparative effectiveness studies specifically designed for LMIC settings, assessing conservative management methods (e.g., pelvic floor muscle training, pessaries, lifestyle interventions) against surgical interventions such as native tissue repairs. Most existing high-quality evidence derives from high-income countries, limiting generalizability due to significant differences in healthcare infrastructure, patient populations, cultural acceptability, and resource availability. As a result, evidence-based guidelines specific to LMIC contexts remain elusive, reinforcing the need for robust comparative trials conducted within culturally and economically similar environments. Large epidemiological studies are essential to achieve meaningful insight into long-term outcomes and real-world effectiveness. Alternatively, the systematic use of electronic healthcare records (EHRs) can enable continuous, longitudinal ‘living-analyses' conducted over at least 20 years, with interim evaluations every 24–36 months, to inform contextually relevant policy and practice in LMICs. Absence of clear national guidelines for prolapse screening, management, and postpartum prevention.

National clinical guidelines that systematically incorporate prolapse prevention, screening, diagnosis, conservative management, and surgical care are virtually absent in many LMICs (37). This lack of standardised guidelines leads to variability in practice, missed opportunities for early prevention or intervention, and reduced overall quality of care. Including clear guidance on prolapse assessment within routine postpartum and reproductive health services could significantly improve early identification and prevention, representing an important clinical and policy gap. Challenges and Opportunities for Prevention and Program Implementation (see Supplementary Table S3 in the supplimentary).

Urgent research priorities identified for addressing cystocele effectively in LMICS include the following, as outlined in Supplementary Table S4 in the supplementary.

## Conclusion

Cystocele represents a widespread yet severely neglected women's health issue, disproportionately impacting women across Africa, Asia, and the Middle East. Despite its' high prevalence and significant impact on physical, psychological, and psycho-social functioning, cystocele remains under-recognised in global and national women's health agendas. Successfully addressing this condition requires comprehensive strategies that bridge clinical practice gaps, robust epidemiological and implementation research, targeted sociocultural interventions, and sustained policy commitment.

Enhancing early detection through systematic screening and surveillance studies, improving healthcare worker training in conservative management methods, and expanding access to skilled urogynaecological care are urgent clinical priorities. Concurrently, rigorous epidemiological and comparative effectiveness research tailored specifically to LMIC contexts will help establish robust evidence-based practices, guiding culturally appropriate, scalable solutions.

Critically, overcoming sociocultural stigma through targeted community awareness, education campaigns, and engagement of key community influencers is essential to empowering women and facilitating timely care-seeking behaviours. Additionally, integrating prolapse care into broader maternal and reproductive health frameworks, supported by sustainable financing and national policy guidelines, will provide structural stability and ensure long-term program effectiveness.

Ultimately, prioritising cystocoele as a public health and human rights issue hold immense potential to transform the lives of millions of women. By investing strategically in culturally sensitive interventions, evidence-driven practices, and equitable health systems, affected regions can achieve substantial improvements in women's health, dignity, and societal participation.

## Data Availability

The original contributions presented in the study are included in the article/Supplementary Material, further inquiries can be directed to the corresponding author.
