# Supplementary material for: Bladder prolapse (cystocele) among African, Asian, and Middle Eastern women: a clinical and socio-cultural perspective
Source: Front Glob Womens Health. 2025 Nov 24;6:1696375. doi: 10.3389/fgwh.2025.1696375 (PMC12682755; doi:10.3389/fgwh.2025.1696375)
Supplement: Supplementary file 1 [file Supplementaryfile1.docx]

Supplementary

**Table 1: Risk Factors in the Aetiology of Bladder Prolapse (Cystocoele)**

| **Risk Factor** | **Description** |
| --- | --- |
| **High parity and closely spaced pregnancies** | Repeated childbirth significantly increases cumulative pelvic floor damage, particularly in settings where obstetric care and postpartum recovery services are inadequate [8, 12]. Inadequate access to skilled birth attendants, delayed recognition of obstetric trauma, and insufficient postpartum rehabilitation exacerbate pelvic floor weakening over time. This cumulative damage contributes to a higher risk of conditions such as cystocele.[[8](#_ENREF_8), [12](#_ENREF_12)]. |
| **Early childbirth** | Teenage pregnancies expose immature pelvic structures to trauma, with a longer lifetime risk of pelvic floor dysfunction [[9](#_ENREF_9), [13](#_ENREF_13)]. |
| **Traumatic and prolonged labour** | Unattended or poorly managed labours are a major cause of significant pelvic trauma, leading to increased risk of cystocele development [14]. Prolonged obstructed labour, excessive use of force during delivery, and lack of timely medical interventions can result in direct injury to the pelvic floor muscles, connective tissues, and supporting structures. Such trauma weakens the anatomical support of the bladder and urethra, predisposing women to symptomatic pelvic organ prolapse later in life, particularly during the hormonal changes of . |
| **Postpartum physical labour** | Early resumption of heavy physical activity post-delivery hinders pelvic floor recovery, raising prolapse risk [[12](#_ENREF_12), [13](#_ENREF_13)]. |
| **Poor nutrition and anaemia** | Nutritional deficits, particularly deficiencies in essential vitamins and minerals such as vitamin C, zinc, and protein, impair connective tissue strength and resilience, increasing vulnerability to pelvic floor disorders [12]. These deficiencies are especially prevalent in rural and low-resource settings, where limited access to balanced diets exacerbates maternal health risks. Poor nutritional status during critical periods such as pregnancy, childbirth, and menopause compromises the body's ability to repair and maintain the structural integrity of pelvic tissues, thereby elevating the risk of conditions like cystocele. |
| **Chronic respiratory conditions and constipation** | Persistent coughing, often resulting from chronic exposure to indoor air pollution, respiratory infections, or smoking, as well as habitual straining due to chronic constipation, significantly increases intra-abdominal pressure [18]. This sustained pressure places repeated mechanical stress on the pelvic floor muscles, ligaments, and connective tissues, accelerating their weakening over time. In settings where respiratory illnesses and gastrointestinal issues are common and under-treated, these factors contribute substantially to the development and progression of pelvic organ prolapse, including cystocele, particularly in women already vulnerable due to hormonal changes and previous obstetric trauma. |
| **Obesity** | Excessive weight gain, particularly central obesity, raises intra-abdominal pressure, thereby increasing mechanical strain on the pelvic floor structures and heightening the risk of pelvic organ descent, including cystocele [18]. The added pressure from excess body mass compromises the ability of pelvic muscles and connective tissues to maintain organ support, especially when compounded by age-related tissue weakening and prior obstetric injuries. Furthermore, obesity is often associated with other risk factors such as chronic coughing, metabolic disorders, and physical inactivity, all of which can exacerbate pelvic floor dysfunction and worsen the severity of prolapse over time [[19](#_ENREF_19)]. |
| **Menopause and ageing** | Declining estrogenic post-menopause reduces pelvic tissue elasticity and support, especially with advancing age. Women in the menopausal age group are often under prioritised in healthcare despite their critical societal roles [57]. This neglect contributes to significant gaps in addressing conditions such as bladder prolapse, and its consequent impact on urinary, sexual and mental health, which arises during menopause due to hormonal changes. The under recognition of midlife women's health needs results in delayed diagnoses, inadequate support, and limited access to effective interventions, even though such conditions are largely preventable [[13](#_ENREF_13), [18](#_ENREF_18)].. |
| **Genetic predisposition** | Familial or ethnic differences in connective tissue strength may predispose certain populations to higher prolapse risk, though research remains limited [[13](#_ENREF_13)]. |

Table 2: Treatment and Management Strategies in Bladder Prolapse Management

| **Type** | **Intervention** | **Description and Benefits** | **Challenges/ Limitations** |
| --- | --- | --- | --- |
| **Conservative** | **Pelvic Floor Muscle Training (PFMT)** | Structured exercises (Kegel exercises) to strengthen pelvic support structures; effective for early stage prolapse and mild symptoms. | Limited awareness, insufficient provider training, lack of patient education in LMICs [[23](#_ENREF_23)]. |
|  | **Vaginal Pessaries** | Mechanical support device inserted into the vagina; alleviates symptoms in moderate cases and improves quality of life. | Underutilization due to cultural hesitance, limited provider expertise, and supply issues in LMICs [[24](#_ENREF_24)]. |
|  | **Lifestyle Modifications** | Counselling on reducing heavy lifting, managing constipation/respiratory conditions, weight control, and improving nutrition for tissue health. | Requires sustained behavioural change and long-term follow-up; may be deprioritized in low-resource settings [[22](#_ENREF_22)]. |
|  | **Topical Oestrogen Therapy** | Applied in postmenopausal women to improve vaginal elasticity and pessary tolerance; it may relieve mild symptoms. | Limited availability and awareness; benefits mostly for postmenopausal women [[25](#_ENREF_25)]. |
| **Surgical** | **Anterior Colporrhaphy** | Native tissue vaginal repair of the anterior wall; first-line surgical treatment for symptomatic or advanced prolapse. | Requires surgical expertise; long-term efficacy may vary [[26](#_ENREF_26)]. |
|  | **Combined Repair with Hysterectomy** | Used in multi-compartment or uterine prolapse; anterior/posterior repair with vaginal hysterectomy. | Complex procedure; needs specialist training and postoperative care [[26](#_ENREF_26)]. |
|  | **Mesh Repair (declining use)** | Formerly used synthetic mesh for support; now discouraged due to complications such as erosion and chronic pelvic pain. | Global decline due to adverse effects; replaced by native tissue repairs [[27](#_ENREF_27)]. |
|  | **Surgical Camps/Outreach Programs** | Mobile or temporary surgical setups (e.g., Nepal, Ethiopia) to increase access in underserved regions. | In remote settings, postoperative follow-up, long-term monitoring, and complication management remain difficult [[28](#_ENREF_28)]. |

Table 3: Challenges and Opportunities for Prevention and Program Implementation Future

| **Category** | **Issue** | **Description** |
| --- | --- | --- |
| **Challenges** | **Lack of Integration in Maternal Health Services** | Prolapse prevention is rarely addressed in existing maternal health programs, which prioritize maternal/neonatal mortality, and missing opportunities for postpartum pelvic floor care. |
|  | **Limited Access to Specialized Treatment** | Geographic isolation, financial barriers, weak infrastructure, and shortage of trained specialists restrict timely diagnosis and surgical care, especially in rural settings. |
|  | **Sociocultural Stigma and Delayed Care-Seeking** | Cultural shame, misinformation, and gender inequalities delay help-seeking, worsening disease progression and increasing emotional distress. |
| **Opportunities** | **Routine Screening in Maternal Healthcare** | Prolapse screening, risk-factor education, and early conservative interventions can be embedded into antenatal and postpartum services to enable early detection and care. [[52](#_ENREF_52)] |
|  | **Provider Training, Task-Shifting, and Pessary Access** | Training midwives and nurses in prolapse care and pessary fitting can decentralize services and improve access in low-resource areas. [[53](#_ENREF_53)] |
|  | **Culturally Sensitive Community Education** | Public health campaigns can reduce stigma, raise awareness about treatment options, and encourage women to seek care earlier. [[54](#_ENREF_54)] |
|  | **Health System Strengthening and Financing** | Embedding prolapse care into broader women’s health and human rights agendas, with dedicated funding, can ensure sustainability and quality of care. [[55](#_ENREF_55)] |

Table 4: Urgent research priorities identified for addressing cystocele effectively in LMICS

| **Research Priority** | **Description** |
| --- | --- |
| **Clinical Epidemiological Studies** | Population-based studies are essential to accurately determine the true prevalence, risk factors, and health outcomes of bladder prolapse (cystocele) across diverse regional settings. Current data, largely hospital-based, underrepresents the burden of disease, particularly among rural and marginalised populations. Most LMICs do not have healthcare organisations with electronic healthcare records. Comprehensive epidemiological research needs to be conducted using innovative approaches that can guide effective prevention, early detection, and management strategies tailored to local needs.  Key study objectives should include:   - **Prevalence Estimation**   - Use standardized diagnostic methods to assess true prevalence, stratified by age, parity, ethnicity, and location. - **Risk Factor Analysis**   - Identify contributory factors such as childbirth practices, menopause, obesity, respiratory illnesses, and limited healthcare access. - **Health Outcomes Assessment**   - Measure the physical, psychological, sexual, and social impacts of bladder prolapse on quality of life. - **Cultural and Environmental Contexts**   - Explore the influence of cultural beliefs, traditional practices, and environmental exposures on disease development and care-seeking. - **Equity and Disparities**   - Analyse differences in prevalence and outcomes across socioeconomic and ethnic groups to guide equity-focused interventions. - **Policy and Practice Implications**   - Translate findings into recommendations for integrating prolapse care into reproductive health programs and awareness campaigns.   . |
| **Comparative Effectiveness Trials and other forms of studies** | Evaluating the outcomes of conservative treatments, such as pelvic floor muscle training (PFMT) and pessary use, versus surgical interventions for pelvic organ prolapse is critical, particularly in resource-limited and culturally diverse populations. Conservative approaches may offer affordable, less invasive options that align better with local preferences, but surgical interventions remain essential for advanced cases. Comparative assessments are needed to guide evidence-based, culturally appropriate care.  Key evaluation priorities include:   - **Clinical Effectiveness**   - Compare symptom improvement, quality of life, and recurrence rates between conservative and surgical treatments. - **Cultural Acceptability**   - Assess women's preferences, cultural beliefs, and barriers to uptake for both non-surgical and surgical options. - **Adherence and Sustainability**   - Evaluate long-term adherence to PFMT programs and pessary use and identify factors influencing sustained engagement. - **Safety and Complication Rates**   - Monitor adverse events associated with conservative devices and surgical procedures, particularly where follow-up services are limited. - **Cost-Effectiveness**   - Compare the economic viability of conservative management versus surgery, including healthcare system and patient-level costs. - **Patient-Cantered Outcomes**   - Measure satisfaction, functional outcomes, psychological well-being, and impact on daily life. |
| **Implementation Research** | Integrating prolapse screening and treatment into existing maternal and reproductive health services offers a strategic opportunity to reach women early, reduce stigma, and improve long-term outcomes. Given the shared risk factors between childbirth, reproductive health events, and pelvic floor dysfunction, embedding prolapse care into routine maternal services is both logical and potentially cost-effective. However, careful exploration of the feasibility, acceptability, and effectiveness of such integration is necessary to ensure successful implementation, particularly in resource-constrained settings.  Key considerations for integration include:   - **Feasibility Assessment**   - Evaluate the availability of trained personnel, diagnostic tools (e.g., pelvic examination capacity), and referral systems within existing maternal health programs.   - Assess the infrastructure needs, including privacy standards for examinations and the potential workload impact on healthcare providers. - **Acceptability to Women and Communities**   - Conduct qualitative research (e.g., focus groups, interviews) to understand women’s attitudes toward prolapse screening during antenatal, postnatal, and family planning visits.   - Explore community norms around pelvic health discussions to identify culturally appropriate ways to offer screening without increasing stigma. - **Acceptability to Healthcare Providers**   - Survey and train maternal and reproductive health providers to assess their willingness, comfort level, and capacity to incorporate prolapse assessment and education into routine care. - **Effectiveness and Impact Evaluation**   - Pilot integrated screening models and measure outcomes such as early prolapse detection rates, referral uptake, treatment initiation, and patient satisfaction.   - Monitor potential unintended consequences, such as increased anxiety among women or overburdening of already stretched maternal health services. - **Cost-Effectiveness Analysis**   - Compare the costs and benefits of integrated prolapse screening versus stand-alone pelvic health services, considering long-term savings from early diagnosis and prevention of severe morbidity. - **Policy and Programmatic Adaptations**   - Advocate for national reproductive and maternal health guidelines to explicitly include pelvic floor health, ensuring that screening and basic management are standardized and scaled. |
| **Inclusion of cultural adaptations** | Cultural adaptations are essential to ensure that healthcare approaches for pelvic floor disorders are inclusive, accessible, and effective, particularly in low- and middle-income countries (LMICs), where populations are often multi-racial, multi-ethnic, and shaped by diverse cultural traditions. Designing interventions without cultural sensitivity risks alienating key groups, perpetuating stigma, and reducing engagement with healthcare services. Therefore, public health initiatives, clinical care models, and community education programs must be culturally adapted to reflect the values, languages, health beliefs, and traditional practices of the populations they aim to serve.  Key cultural adaptation strategies include:   - **Community Engagement and Co-creation** Involve women, families, and community leaders in the design and delivery of pelvic health programs to ensure relevance and acceptance. - **Linguistic Inclusivity** Provide health education materials and clinical services in multiple local languages to accommodate linguistic diversity and improve comprehension. - **Respect for Traditional Health Beliefs** Recognize and respectfully integrate or address traditional understandings of health, illness, childbirth, and aging, avoiding direct confrontation where possible. - **Training of Culturally Competent Healthcare Providers** Equip healthcare workers with cultural competence training to improve sensitivity, communication, and trust-building with diverse patient populations. - **Representation in Health Messaging** Use diverse imagery, narratives, and role models in health promotion campaigns to reflect the multi-ethnic composition of the target audience. - **Flexibility in Service Delivery Models** Adapt clinic structures, appointment systems, and outreach models (e.g., community-based care, mobile clinics) to accommodate cultural norms around gender roles, mobility, and family obligations. - **Support for Gender-Sensitive Access** Address barriers that women face in male-dominated health systems by ensuring availability of female healthcare providers where culturally appropriate. |
| **Development of National Guidelines** | - **Create stigma-free healthcare environments** Foster respectful, non-judgmental settings where women feel empowered to discuss pelvic and reproductive health concerns openly. - **Prioritise women's health across the life course** Address pelvic organ prolapse, urinary incontinence, and sexual dysfunction as important health issues at all stages of life, not limited to menopause. - **Recognise critical transition periods** Pay particular attention to high-risk phases such as postpartum recovery and menopause, when pelvic floor vulnerability is heightened. - **Raise public awareness and dismantle stigma** Launch educational campaigns and community initiatives to normalize discussions about pelvic health and encourage timely care-seeking. - **Develop context-specific clinical guidelines** Base prevention, screening, management, surgical intervention, and follow-up protocols on local epidemiological evidence and cultural context. - **Ensure culturally sensitive and equitable care pathways** Design healthcare services that are accessible, affordable, and responsive to the diverse needs of women in different regions and communities. |
